# Primary Renal Lymphoma: A Rare Cause of Bilateral Renal Enlargement and Acute Renal Failure in a Patient With Rheumatoid Arthritis

**DOI:** 10.7759/cureus.63870

**Published:** 2024-07-04

**Authors:** Parisa Aijaz, Muhammad A Niazi, James P Ferrick, Christopher H Goss, Amir Kamran

**Affiliations:** 1 Internal Medicine, Charleston Area Medical Center, Charleston, USA; 2 Internal Medicine, Dow University of Health Sciences, Karachi, PAK; 3 Hematology and Medical Oncology, Charleston Area Medical Center, Charleston, USA

**Keywords:** onco-nephrology, renal insufficiency, non-hodgkin, lymphoma, case reports

## Abstract

Primary renal lymphoma (PRL) is a rare non-Hodgkin's lymphoma (NHL) involving the kidneys without evidence of extra-renal involvement. We describe a 66-year-old female who presented with bilateral pleural effusions, and acute renal failure and was diagnosed with primary renal diffuse large B-cell lymphoma (DLBCL). She presented with shortness of breath due to bilateral pleural effusions and acute renal failure. Computed tomography (CT) of the chest reported bilateral pleural effusions. Thoracocentesis and subsequent fluid analysis reported non-malignant effusion. Her kidney function worsened during her hospital stay, requiring dialysis. Nonspecific findings such as bilateral renal enlargement on imaging prompted a renal biopsy. Histopathology reported mixed tubulointerstitial atypical lymphocytic CD 20 and BCL-6 positive cell infiltrates, confirming non-Hodgkin diffuse large B-cell lymphoma. Whole-body positron emission tomography/CT (PET/CT) and brain magnetic resonance imaging (MRI) ruled out the involvement of any other organs or lymph nodes, confirming our diagnosis of PRL. She was treated with six cycles of rituximab, cyclophosphamide, doxorubicin, vincristine, and prednisone (R-CHOP). Her kidney function recovered fully and remained normal at the one-year follow-up. We highlight the importance of recognizing PRL as an underlying cause of renal failure and its association with autoimmune diseases. Prompt investigation with timely diagnosis and treatment can result in improved morbidity and mortality in these patients.

## Introduction

Non-Hodgkin's lymphoma (NHL) is a cancer of lymphoid origin, accounting for 4.35% of cancers in the United States [1]. NHL is associated with viruses, immunodeficiency states, and autoimmune disorders such as Sjogren's syndrome and rheumatoid arthritis [1]. Primary renal lymphoma (PRL) is defined as NHL involving the kidneys without evidence of extra-renal involvement [2]. It is a rare occurrence and comprises < 1% of all extranodal NHL [3]. Although previously challenged, the diagnosis of PRL is well established with an increasing number of reported cases [3]. It is still very rare, with less than a hundred reported cases to date [3]. Diffuse large B-cell lymphoma (DLBCL) is the most common histological subtype of PRL [2,3]. PRL more commonly occurs in the elderly with a median age of 70-72 years. It is more common in males as compared to females, with a reported ratio of 1.6:1 [2,3]. The etiology is not fully understood and more research needs to be done to further investigate the underlying mechanisms. Patients present with non-specific findings such as hematuria, flank pain, and renal failure [2]. We describe a 66-year-old female presenting with renal failure who was diagnosed with primary renal diffuse large B-cell lymphoma (DLBCL) and was successfully treated with rituximab, cyclophosphamide, doxorubicin, vincristine, and prednisone (R-CHOP) resulting in a complete renal recovery. 

## Case presentation

**Figure 1:**
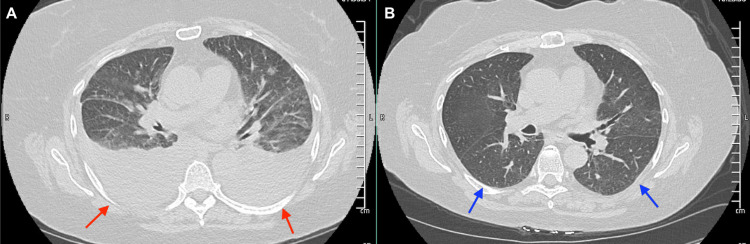
A: Computed tomography of the chest showing bilateral pleural effusions in a patient with renal failure due to PRL (red arrows). B: CT Chest showing resolution of bilateral pleural effusions after treatment (blue arrows). PRL: primary renal lymphoma

**Figure 2:**
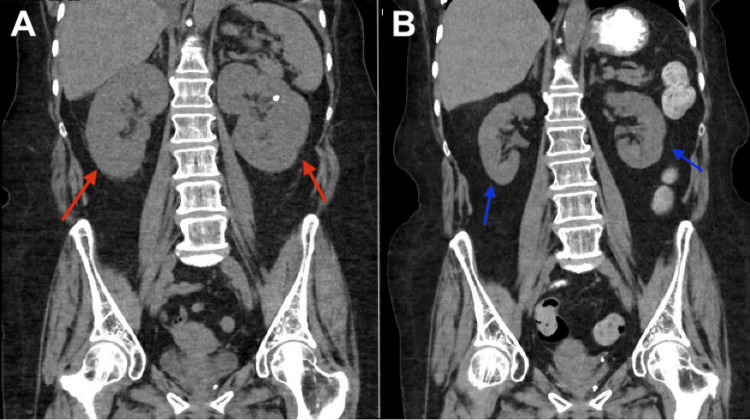
A: Computed tomography of the abdomen showing bilateral renal enlargement in a 66-year-old female diagnosed with PRL (red arrows). B: Improvement of bilateral renal enlargement on CT abdomen after treatment with six cycles of R-CHOP (blue arrows). PRL: primary renal lymphoma; R-CHOP: rituximab, cyclophosphamide, doxorubicin, vincristine, and prednisone

**Figure 3:**
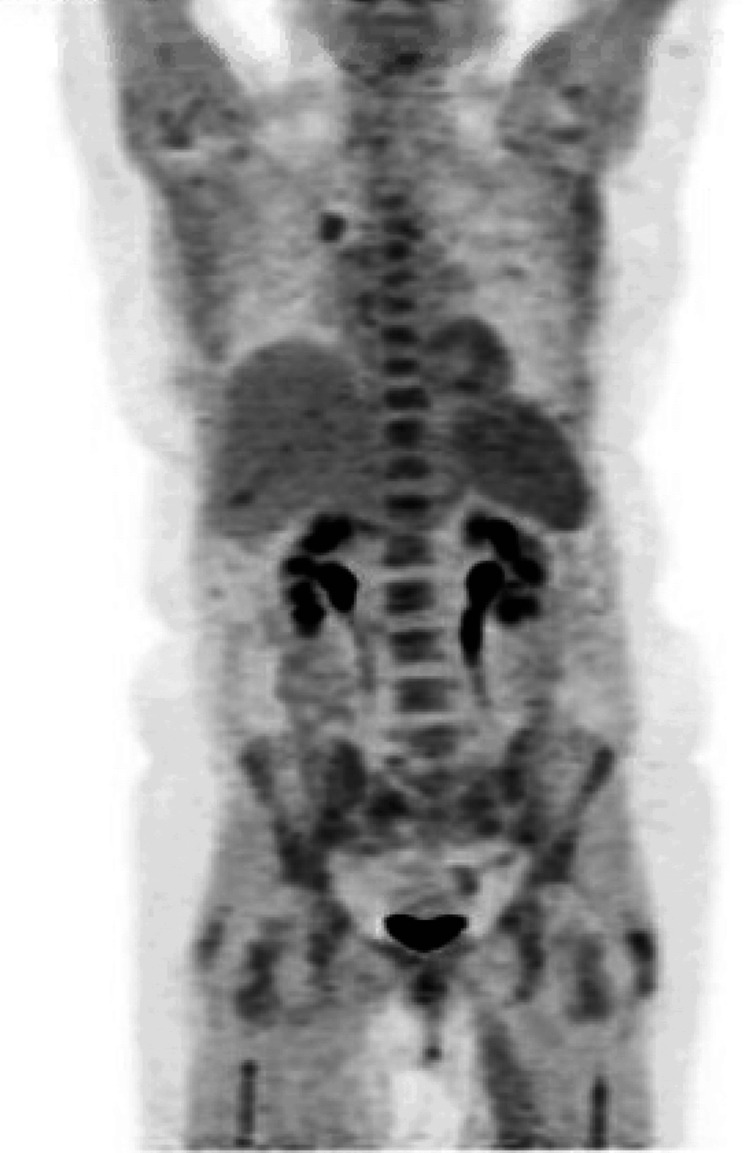
Positron emission tomography/computed tomography with no evidence of extra-renal involvement in a patient with primary renal lymphoma

We present a 66-year-old patient with a past medical history of recurrent urinary tract infections, non-obstructing renal stones, rheumatoid arthritis treated with methotrexate, and hypertension. She presented to the emergency department with shortness of breath and band-like lower chest pain for two days. She denied any fevers, chills, night sweats, or unintentional weight loss. She denied smoking or alcohol consumption. Her chest pain was characteristically non-cardiac. Workup was significant for an elevated creatinine of 1.4 mg/dL (normal (n): 0.6 - 1.2 mg/dL) (baseline normal creatinine), estimated glomerular filtration rate (eGFR) of 35 mL/min/1.73 m2, fractional excretion of sodium (FeNa) 0.6%, and an elevated D-dimer (Table 1). B-type natriuretic peptide (BNP) and echocardiogram were normal. A chest computed tomography (CT) was obtained and reported bilateral pleural effusions (Figure 1A). Pleural fluid analysis was negative for malignancy. CT abdomen and pelvis reported enlarged kidneys bilaterally (Figure 2A), a non-obstructing stone in the lower pole of the right kidney, and no radiologic evidence of hydronephrosis. Her pleural effusions and shortness of breath improved with diuresis; however, her kidney function worsened to a creatinine of 6.8 mg/dL and an eGFR of 6 mL/min/1.73 m2, along with hyperkalemia and oliguria. A left-sided kidney biopsy was performed. She required hemodialysis due to renal failure, seven days after admission. The histopathology reported normocellular glomeruli and mixed tubulointerstitial atypical lymphocytic cell infiltrates indicative of large B-cell lymphoma. Tumor cells were positive for cluster of differentiate (CD) 20 and B-cell lymphoma 6 protein (BCL-6). Congo red staining, immunoglobulin G (IgG), B-cell lymphoma/leukemia-2 (BCL-2), CD-30, and c-MYC were negative. She tested negative for hepatitis and human immunodeficiency virus (HIV). A positive emission tomography/CT (PET/CT) scan did not reveal hepatosplenomegaly or lymphadenopathy (Figure 3), and a brain magnetic resonance imaging (MRI) ruled out metastasis or lesions indicative of lymphoma in the brain. We determined that the patient had PRL. We started treatment with intravenous steroids, which resulted in an improvement in her renal function. Her oliguria resolved and electrolytes normalized. She was treated with systemic chemotherapy with a 21-day cycle of R-CHOP for six cycles. She did not receive maintenance chemotherapy. A follow-up PET scan four months after diagnosis did not reveal any lymph node or extra-nodal involvement. The patient continues to do well with normal renal function one year after treatment. CT abdomen reported resolution of her bilateral renal enlargement (Figure 2B). She has not had an exacerbation of her rheumatoid arthritis after discontinuation of anti-rheumatic treatment.

**Table 1:** Table reporting results of the pertinent workup of a patient diagnosed with primary renal lymphoma PEP: Protein electrophoresis; CD 20: cluster of differentiate 20; BCL 6: B-cell lymphoma 6 protein; BCL 2: B-cell lymphoma 2 protein; BNP: B-type natriuretic peptide; HIV: human immunodeficiency virus

WORKUP	RESULTS	REFERENCE RANGE
Creatinine	6.8 mg/dL before treatment, improved to 0.8 mg/dL after treatment	0.7 – 1.3 mg/dL
Fractional excretion of sodium	0.6%	
B2 microglobulin on PEP	4.91 mcg/ml	1.21 – 2.70 mcg/mL
Alpha-2 globulin on PEP	Alpha-2 globulin 1.4 g/dL	0.6 – 1 g/dL
Positive molecular markers	CD-20, BCL-6	
Negative molecular markers	Congo red staining, IgG, BCL-2, CD-30, c-MYC	
BNP	10.0 pg/mL	0 – 100 pg/mL
Echocardiogram	Normal systolic and diastolic function, normal valves	
Infectious workup	Negative hepatitis B, hepatitis C and HIV	

## Discussion

Recognizing PRL as an underlying etiology of renal engorgement and renal failure can lead to timely diagnosis and potentially curative treatment for renal failure, improving morbidity and mortality. Although kidneys are commonly involved in extra-nodal lymphoma, isolated renal involvement, i.e. PRL is rare (< 1%), with an age-adjusted incidence reported at 0.035-0.053/100,000 in the general population [3,4]. The disease occurs primarily in elderly patients with a median age of 72 years [3]. There also appears to be a male predominance, with male-to-female ratios of 1.6-1.7:1 noted in some studies [4]. Bilateral renal involvement, such as in our patient, is seen in only 10-20% of cases [3]. The most common histological subtype is diffuse large B-cell lymphoma. It arises from germinal or post-germinal cells. Around 70% of the tumor cells are positive for B-cell lymphoma 6 (BCL 6) protein and approximately 80% of tumor cells are positive for B-cell lymphoma/ leukemia 2 protein (BCL-2) [5]

Risk associations are similar to NHL and include viral infections, including EBV, HIV, immunodeficiency states, medications, and autoimmune disorders [6]. Our patient has a history of rheumatoid arthritis (RA) and was on methotrexate which has been associated with an increased risk of NHL, particularly DLBCL. There is debate regarding whether methotrexate poses an independent risk in the increased incidence of NHL in patients with RA [1,7,8]. Studies have reported methotrexate-associated lymphoproliferative disorders including DLBL and classic Hodgkin's lymphoma. Pathophysiology is hypothesized to be the reactivation of latent EBV in about half the patients, whereas, in patients who test negative for EBV, a mechanism has not yet been determined [8-10]. The EBV status of our patient was not checked at the time of diagnosis. 

The pathophysiology of PRL is uncertain. It is speculated that the capsule might initially be involved, leading to tumor extension into the renal parenchyma. Another proposed explanation is that chronic inflammation attracts lymphocytes to the kidney, a theory that might explain the increased incidence in patients with chronic inflammatory states and autoimmune disorders [2]. 

Patients most commonly present with acute renal failure, flank pain, and a mass on imaging [2]. Our patient had a unique presentation with shortness of breath and bilateral pleural effusions, likely secondary to her underlying renal failure. CT imaging findings mostly commonly include multiple nodules bilaterally, occasional solitary mass, or diffuse renal infiltration and engorgement without an identifiable mass, such as our patient [11,12]. However, CT findings can be non-specific, and renal biopsy is the gold standard for diagnosis.

The treatment for PRL is extrapolated from the treatment of NHL [13]. The mainstay is systemic chemotherapy with R-CHOP [13]. Our patient received six cycles of R-CHOP which led to the disease remission and normalization of her kidney function. New treatment modalities have recently been approved. These include allogeneic stem cell transplant, which has been shown to reduce the rate of recurrence [13], and chimeric antigen receptor (CAR)-T cell therapy, which has recently been approved for relapsed or refractory DLBCL [14]. The role of surgery or radiotherapy in the management of PRL hasn’t been defined and is unlikely to improve outcomes [15]. 

The prognosis of PRL is unknown due to the rarity of the disease, however, given the available data, survival does not appear favorable. Overall, the five-year survival rate is only 40-50% [16-18]. Along with the morbidity associated with DLBCL, its treatment with R-CHOP is also associated with significant adverse effects including infertility, heart failure, secondary leukemia, and osteonecrosis [19]. Prognostic factors that portended a poorer outcome included younger age (0-18 years), bilateral PRL, lesions size >10cm, and diffuse renal infiltration involving the renal hilum [16]. Our patient is doing well without radiological or clinical evidence of disease recurrence, or treatment side effects at the one-year follow-up.

## Conclusions

Primary renal lymphoma is a rare cause of renal failure and bilateral renal enlargement. Our case highlights the importance of prompt investigation with renal biopsy in patients with unexplained worsening renal failure. We also highlight the importance of recognizing risk factors, including autoimmune diseases and medications such as methotrexate. Our patient had a history of rheumatoid arthritis being treated with methotrexate. Keeping this association in mind can prompt us to consider lymphoma as a potential etiology for renal failure, facilitating early diagnoses and timely treatment.
